# Robust autoactivation for apoptosis by BAK but not BAX highlights BAK as an important therapeutic target

**DOI:** 10.1038/s41419-020-2463-7

**Published:** 2020-04-23

**Authors:** Sweta Iyer, Rachel T. Uren, Michael A. Dengler, Melissa X. Shi, Etsuko Uno, Jerry M. Adams, Grant Dewson, Ruth M. Kluck

**Affiliations:** 1 Walter and Eliza Hall Institute of Medical Research, 1G Royal Parade, Parkville, VIC 3052 Australia; 2 Department of Medical Biology, The University of Melbourne, Melbourne, VIC 3052 Australia

**Keywords:** Protein folding, Apoptosis, Mitochondria

## Abstract

BAK and BAX, which drive commitment to apoptosis, are activated principally by certain BH3-only proteins that bind them and trigger major rearrangements. One crucial conformation change is exposure of their BH3 domain which allows BAK or BAX to form homodimers, and potentially to autoactivate other BAK and BAX molecules to ensure robust pore formation and cell death. Here, we test whether full-length BAK or mitochondrial BAX that are specifically activated by antibodies can then activate other BAK or BAX molecules. We found that antibody-activated BAK efficiently activated BAK as well as mitochondrial or cytosolic BAX, but antibody-activated BAX unexpectedly proved a poor activator. Notably, autoactivation by BAK involved transient interactions, as BAK and BAX molecules it activated could dissociate and homodimerize. The results suggest that BAK-driven autoactivation may play a substantial role in apoptosis, including recruitment of BAX to the mitochondria. Hence, directly targeting BAK rather than BAX may prove particularly effective in inhibiting unwanted apoptosis, or alternatively, inducing apoptosis in cancer cells.

## Introduction

The BCL-2 family of proteins, which regulates the mitochondrial (or intrinsic) pathway of apoptosis, comprises three subfamilies^1–3^. Signaling for apoptosis is triggered by the BH3-only subset, such as BIM and BID, but its essential mediators are the multidomain proteins BAK and BAX, which, once activated, can generate pores in the mitochondrial outer membrane (MOM) that unleash proteolytic demolition of the cell by the caspases. The prosurvival family members, such as BCL-2, BCL-X_L_, and MCL-1 act by sequestering BH3-only proteins as well as the activated BAK and BAX proteins. The canonical interactions between family members involve a BH3 domain binding to a hydrophobic α2–α5 surface groove on a globular family member^4,5^.

**Fig. 1:**
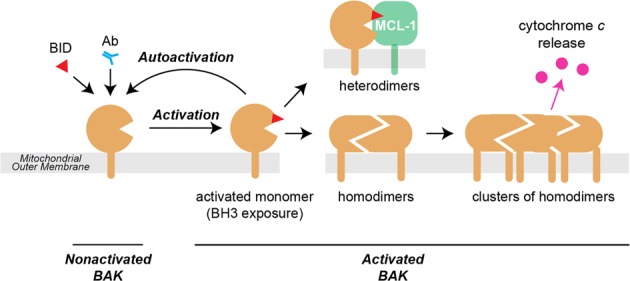
Schematic of BAK activation and autoactivation. In healthy cells, nonactivated BAK is inserted into the mitochondrial outer membrane via its α9 transmembrane domain. Activation can be triggered by BH3-only proteins such as BID (by binding to the α2–α5 groove) or by antibody (binding to the N-terminus of the α1–α2 loop). The major conformation changes that occur during activation include separation of three components—the N-terminal α1–helix, the α2–α5 core, and the α6–α9 latch—leaving the protein with an exposed BH3 domain (α2; red triangle) and a modified α2–α5 hydrophobic groove. (BAK conformation change is also illustrated in an animation of autoactivation in Fig. S6.) Once activated, the BAK monomer can bind to other family members to form either homodimers that promote apoptosis, or heterodimers with a pro-survival relative (here MCL-1) that do not contribute to pore formation. The activated monomer may also activate (autoactivate) additional BAK (or BAX) molecules.

Prior to apoptotic signaling, BAK is inserted in the MOM with its hydrophobic α9 helix forming a transmembrane domain^6,7^, whereas BAX is largely cytosolic with its α9 partially sequestered in a hydrophobic α2–α5 surface groove^8–10^. As depicted in Fig. 1, with the initiation of apoptosis, BAK and BAX are activated by BH3-only proteins. The remarkable ensuing conformation changes include dissociation of the α1 helix, and separation of the α2–α5 core and α6–α8 latch domains which also exposes their BH3 domain (in the α2 helix)^11–15^. The unfolded activated BAK and BAX monomers can then dimerize via reciprocal BH3:groove interactions to form “symmetric homodimers”^12,13,16–22^ that cluster on the MOM to release apoptogenic factors from the mitochondrial intermembrane space^23–25^. Alternatively, the activated BAK and BAX monomers may bind to prosurvival members to form heterodimers that do not participate in pore formation^26–30^.

The activated BAK and BAX monomers are also proposed to activate additional BAK and BAX molecules, a process called auto-oligomerization or autoactivation (Fig. 1)^31–34^. For example, once its BH3 domain is exposed, a BAK or BAX monomer is postulated to act like a BH3-only protein and trigger conformation change in nonactivated BAK or BAX molecules. Autoactivation would act as a feed-forward pathway to enhance the likelihood that all pore-forming proteins in the cell become engaged to ensure cell death. Autoactivation is also proposed as a mechanism to promote BAX translocation from the cytosol^28,32^.

Autoactivation appears plausible as the BAK and BAX BH3 sequences closely resemble those of the activator BH3-only proteins, including four hydrophobic (h1–4) residues and an invariant aspartate residue (Fig. S1a)^12,14,35,36^. Activation sites on the receptor BAK and BAX molecules might include the canonical α2–α5 surface groove^12,14,37^ or a modeled α1/α6 trigger site^32,38–40^ (Fig. S1b). Several studies have shown that BAK and BAX BH3 peptides can activate the full-length proteins in mitochondrial and liposome assays^12,13,36,41^ and when injected into cells^34^. Even stronger activation by the BAK and BAX BH3 domains was evident with chimeras of BID or BIM having their BH3 domain replaced by that of BAK or BAX, probably because the chimeras target to mitochondria^29,36^.

Determining whether full-length activated BAK and BAX anchored at the MOM can activate other BAK or BAX molecules has been more challenging. The concept of BAK autoactivation (or auto-oligomerization) was supported by the ability of an activated BAK mutant (N-terminally truncated BAKΔN36) to trigger oligomerization of endogenous BAK in mitochondria^33^. Evidence of BAX autoactivation derived from experiments in which liposomes that had been partially permeabilized by BAX plus peptide and then fractionated away from excess protein became further permeabilized by addition of full-length BAX^34^. Other studies argue that BAX autoactivation occurs via the proposed α1/α6 trigger site, as a stapled BAX BH3 peptide targeted that site^32^. The concept of activation between full-length BAK and BAX proteins has not been tested^42^, in part because activators that can discriminate BAK and BAX have not been available.

We recently reported that antibodies can selectively activate BAK or the mitochondria-targeted BAX variant S184L^43,44^. For example, the 7D10 antibody binds to the N-terminus of the BAK α1–α2 loop and triggers conformation changes and oligomerization of BAK. Antibodies such as 3C10 that bind to the same region in BAX activate the mitochondrial S184L variant via the same mechanism. Unexpectedly, the BAX antibodies instead inhibit wild-type (cytosolic) BAX by allosterically sequestering its α9 transmembrane domain within its surface groove, preventing BAX translocation to mitochondria^43,44^.

Here, we have exploited antibody specificity for BAK or BAX to test for autoactivation by the full-length BAK and BAX proteins on mitochondria. Pairing different forms of BAK and BAX revealed that antibody-activated BAK could activate BAK, mitochondrial BAX and cytosolic BAX, whereas activated BAX unexpectedly proved a poor activator of all three targets. Curiously, the BAX groove mutant, R109D, proved a robust activator. Despite the potential of reciprocal BH3:groove interactions to simply generate stable dimers, autoactivation by BAK involved a transient interaction, as the targeted (activated) proteins could homodimerize. The robust autoactivation we observe with BAK indicates that developing direct activators of BAK (rather than of BAX) may best recruit additional pore-forming BAK and BAX molecules to induce robust apoptosis in cancer cells. Conversely, direct inhibitors of BAK, by precluding autoactivation, may prove particularly effective in inhibiting unwanted apoptosis.

## Materials and methods

### Cloning and expression of BAK and BAX variants

For expression in cells, BAK and BAX mutants were generated by site-directed mutagenesis and overlap extension PCR, with BamHI and XhoI used for cloning into the pMIH hygromycin selectable vector, and introduced mutations confirmed by DNA sequencing, as described^17^. The variants were then stably expressed in SV40-immortalized *Bak*^*−/*−^*Bax*^*−/−*^ mouse embryonic fibroblasts (MEF), and polyclonal populations of green fluorescent protein-positive cells or hygromycin-resistant MEF selected and cultured as described^17^.

Recombinant BAX^R109D^ and BAX^R34A^ were generated by site-directed mutagenesis of human wild-type or cys-null BAX, respectively^38,44^, and the recombinant wild-type and mutant BAX proteins expressed and purified as described^9^.

### Preparation of mitochondrial fractions from MEF and mouse liver

Mitochondria-enriched membrane fractions from MEF were generated by first resuspending cells at 1 × 10^7^ ml^−1^ in MELB buffer (93.5 mM sucrose, 20 mM HEPES, pH 7.4, 2.5 mM MgCl_2_ and 100 mM KCl) supplemented with Complete Protease Inhibitor cocktail (Roche). Cell membranes were then permeabilized by addition of 0.025% w/v digitonin and incubation on ice for 10 min, followed by centrifugation at 13,000*g* for 5 min to separate the supernatant (cytosolic) and pellet (mitochondria-enriched membrane) fractions. Membrane fractions were resuspended in MELB buffer supplemented with Complete Protease Inhibitor cocktail as above. Mouse liver mitochondria (MLM) were prepared from wild-type or *Bak*^−/−^ C57BL/6 wild-type mice as described^36^.

### Assessment of BAX membrane insertion, BAK and BAX conformation change and oligomerization

Membrane insertion of BAX (expressed in cells or recombinant protein added to MLM) was assessed by sodium carbonate extraction, as described^38^. BAK and BAX conformation change was assessed by limited proteolysis, as described^45^. Cyseine linkage was used to assess oligomerization of BAK and of BAX-S184L, as described^45^.

### Mitochondrial incubations and cytochrome *c* release assays

For activation of BAK or BAX-S184L in permeabilized MEF, membrane fractions (50 μl) were incubated with 100 nM caspase-8-cleaved human Bid (cBID)^46^ or with the indicated antibody (0.1 mg/ml) for 30 min at 30 °C. The 7D10 and 3C10 antibodies are rat monoclonal antibodies generated in house, as previously described^43^. The 7D10 single chain variable fragment (scFv) was kindly generated by Commonwealth Serum Laboratories, Melbourne. For incubations based on mitochondria from mouse liver, MLM were diluted to 1 mg/ml in MELB and supplemented with the indicated concentrations of recombinant human BAX variants and cBID, and samples incubated for 1 h at 37 °C. Stock solutions of recombinant BCL-2 proteins were diluted in MELB + 1% bovine serum albumin to prevent adsorption to plasticware as described^47^. To monitor cytochrome *c* release from mitochondria, reactions were spun at 13,000*g* (10,000*g* for MLMs) and the supernatant and pellet fractions immunoblotted for cytochrome *c*.

### Co-immunoprecipitation

Membrane fractions (at least 2.5 × 10^6^ cells per treatment) were solubilized with 1% w/v digitonin in lysis buffer (20 mM Tris, 135 mM NaCl, 1.5 mM MgCl_2_, 1 mM EGTA, 10% glycerol, pH 7.4) for 1 h on ice. Lysates were centrifuged at 13,000*g* for 5 min and supernatants collected. (No pre-clearing step with Protein G sepharose was performed because the 7D10 and 3C10 antibodies had been added for activation.) Solubilized samples were added to Protein G sepharose, and, where indicated, also supplemented with 4 μg conformation-specific BAK (14–36) or BAX (6A7) antibody and incubated for 1–2 h at 4 °C. Unbound proteins were collected and the resin washed with lysis buffer containing up to 0.1% w/v digitonin. Immunoprecipitated proteins (IP) were eluted by boiling in sample buffer, and together with unbound and total lysates (input), were immunoblotted for BAK and BAX as indicated. To minimize signals from antibody light chains in western blots, heavy chain-specific horseradish peroxidase (HRP)-conjugated goat anti-rabbit and anti-mouse IgG was used as secondary antibody.

### SDS-PAGE and western blotting

Samples were resolved by sodium dodecyl sulfate polyacrylamide gel electrophoresis (SDS-PAGE) (Bio-Rad or Invitrogen NuPAGE Bis–Tris for limited proteolysis) and transferred to 0.22 μm nitrocellulose or polyvinylidene fluoride membranes. Primary antibodies included rabbit polyclonal anti-BAK aa23–38 (1:5000, Sigma #B5897, RRID:AB_258581), anti-BAK NT (1:2,000, Millipore #06-536, RRID:AB_310159), anti-BAX NT (1:1000, Millipore #ABC11, RRID:AB_310143), rat monoclonal anti-BAK (clone 4B5, in-house), anti-BAX (clone 49F9, in-house), mouse monoclonal anti-BAX clone 3 (1:2000, BD Pharmingen #BDB610982, RRID:AB_398295), anti-cytochrome *c* (1:2000, BD Pharmingen #556433, RRID: AB_396417) and anti-FLAG M2 (1:2,000, Millipore #F1804, RRID: AB_262044)). Detection was achieved using HRP-conjugated anti-rabbit (1:5000, Southern Biotech #4010-05, RRID: AB_2632593), anti-rat (1:5000, Southern Biotech #3010-05, RRID: AB_2795801) and anti-mouse (1:2000, Southern Biotech #1010-05, RRID: AB_2728714) secondary antibodies. To avoid signals from antibody light chains in western blots, heavy chain-specific HRP-conjugated goat anti-rabbit IgG (1:5000, Southern Biotech #4041-05, RRID: AB_2795946), and goat anti-rat IgG (1:5000, Southern Biotech #3030-05, AB_2716837) were also used. Proteins were visualized by Luminata Forte Western HRP substrate (Millipore #WBLUF0500) on a ChemiDoc XRS + System, and images processed with ImageLab Software (Bio-Rad).

## Results

To test for autoactivation between full-length BAK and BAX proteins, pairs of the BAK and BAX variants were co-expressed or combined (Table S1) and stimulated with an antibody that directly activates only one of the two proteins. We note that “activation” is used here to denote the early structural unfolding of BAK and BAX to expose the BH3 domain, rather than the final functional step of pore formation (see Fig. 1). “Autoactivation” occurs between BAK and BAK, BAX and BAX, or BAK and BAX molecules, and is distinct from “spontaneous activation”.

### Antibody-activated BAK activates BAK^G51C^

**Fig. 2:**
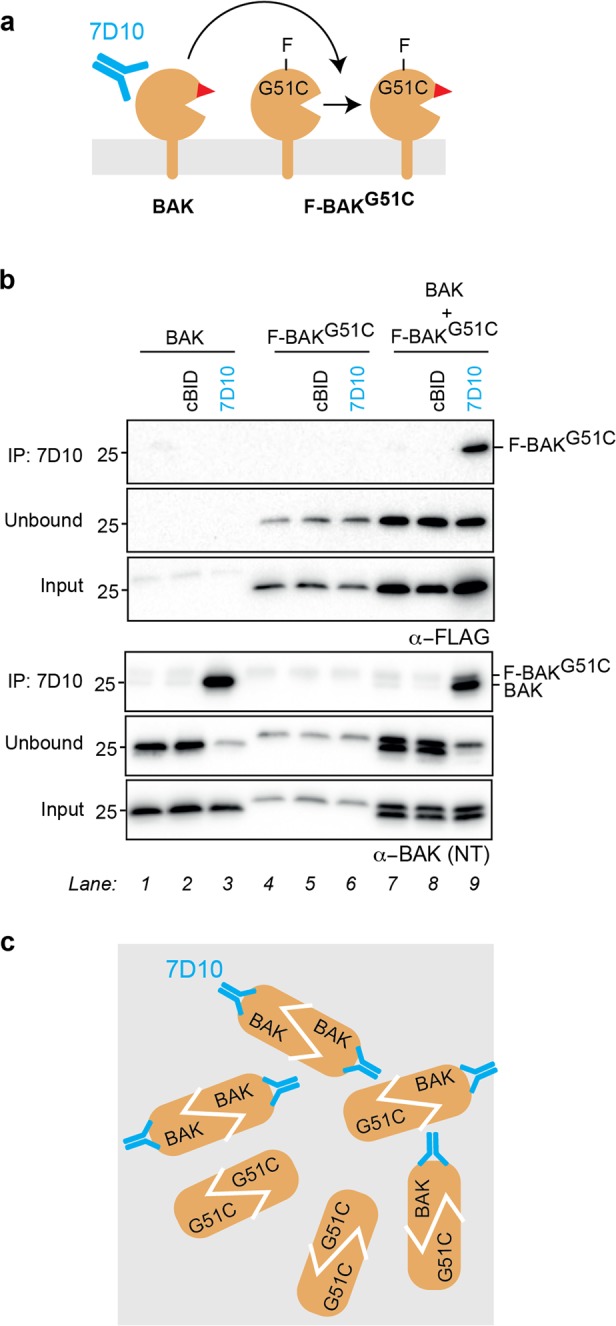
Antibody-activated BAK activates BAK^G51C^. **a** Schematic of two BAK variants (BAK and F-BAK^G51C^) co-expressed in mitochondria and then incubated with the 7D10 antibody to directly activate BAK and expose its BH3 domain (red triangle). 7D10 cannot bind F-BAK^G51C^. **b** Co-immunoprecipitation shows that F-BAK^G51C^ is activated by the 7D10 antibody if BAK is also present. Two BAK variants were expressed individually or together in *Bax*^−*/−*^*Bak*^*−/−*^ mouse embryonic fibroblasts (MEF), and membrane fractions incubated with the 7D10 antibody to activate (wild-type) BAK. As positive controls, aliquots were also incubated with cBID to activate both forms of BAK. Samples were immunoprecipitated using the 7D10 antibody, and immunoblotted for FLAG (upper panels) or BAK (lower panels). Data are representative of two independent experiments. **c** Schematic of BAK dimers captured by the 7D10 antibody. Activated BAK and activated F-BAK^G51C^ associate to form homodimers, some of which 7D10 can immunoprecipitate.

To test for autoactivation of BAK by BAK, as depicted in Fig. 2a, we exploited the ability of the 7D10 antibody to activate wild-type human BAK but not the G51C variant, in which the 7D10 epitope is mutated^43^. The two proteins were stably expressed either individually or together in *Bax*^*−/−*^*Bak*^*−/−*^ MEF. The BAK^G51C^ variant was distinguished by an N-terminal FLAG-tag (i.e., F-BAK^G51C^). The cells were permeabilized and the membrane fractions incubated with cBID (caspase-8 cleaved BID) to activate both wild-type BAK and F-BAK^G51C^, or with the 7D10 antibody to directly activate only wild-type BAK. Activation of the co-expressed F-BAK^G51C^ was then assessed by three methods.

We first assessed the ability of F-BAK^G51C^ to form dimers with 7D10-activated BAK, as detected by immunoprecipitation with 7D10. As expected, when the BAK variants were expressed individually and stimulated with 7D10 antibody, 7D10 precipitated most of the wild-type BAK (Fig. 2b, lower panels, lane 3), but not F-BAK^G51C^ (lane 6). Importantly, with co-expressed WT BAK and F-BAK^G51C^, 7D10 also captured F-BAK^G51C^ (Fig. 2b, upper and lower panels, lane 9), indicating complexes of activated wild-type BAK and F-BAK^G51C^ (Fig. 2c).

A second set of experiments involved immunoprecipitation with a rabbit antibody (14–36) that recognizes the activated forms of both wild-type BAK and F-BAK^G51C^; it was added to all samples at the end of the incubations (Fig. S2a). Again, F-BAK^G51C^ was immunoprecipitated from 7D10-treated samples only if wild-type BAK was also present (Fig. S2b, lane 9 vs. 6). Notably, as nearly all the F-BAK^G51C^ was immunoprecipitated by the 14–36 antibody, its activation was nearly complete. Thus, the autoactivation by antibody-activated BAK was robust.

Finally, we assessed F-BAK^G51C^ activation using disulfide linkage to detect oligomerized proteins (Fig. S2b). When 7D10 was incubated with co-expressed wild-type BAK (C14 and C166) and F-BAK^G51C^, linkage generated three distinct 2× complexes (Fig. S2b, lane 6). Based on size, the two upper complexes contain the larger FLAG-tagged F-BAK^G51C^, indicating that it had become autoactivated by wild-type BAK and could be linked to either activated BAK or activated F-BAK^G51C^. Autoactivation was again near-complete, as most F-BAK^G51C^ could be linked into 2× complexes (Fig. S2b, lane 6). In summary, three methods of detecting activated F-BAK^G51C^ (co-immunoprecipitation, immunoprecipitation, and cysteine linkage) showed that 7D10-activated BAK efficiently autoactivated BAK^G51C^.

### Antibody-activated BAK can activate mitochondrial BAX

**Fig. 3:**
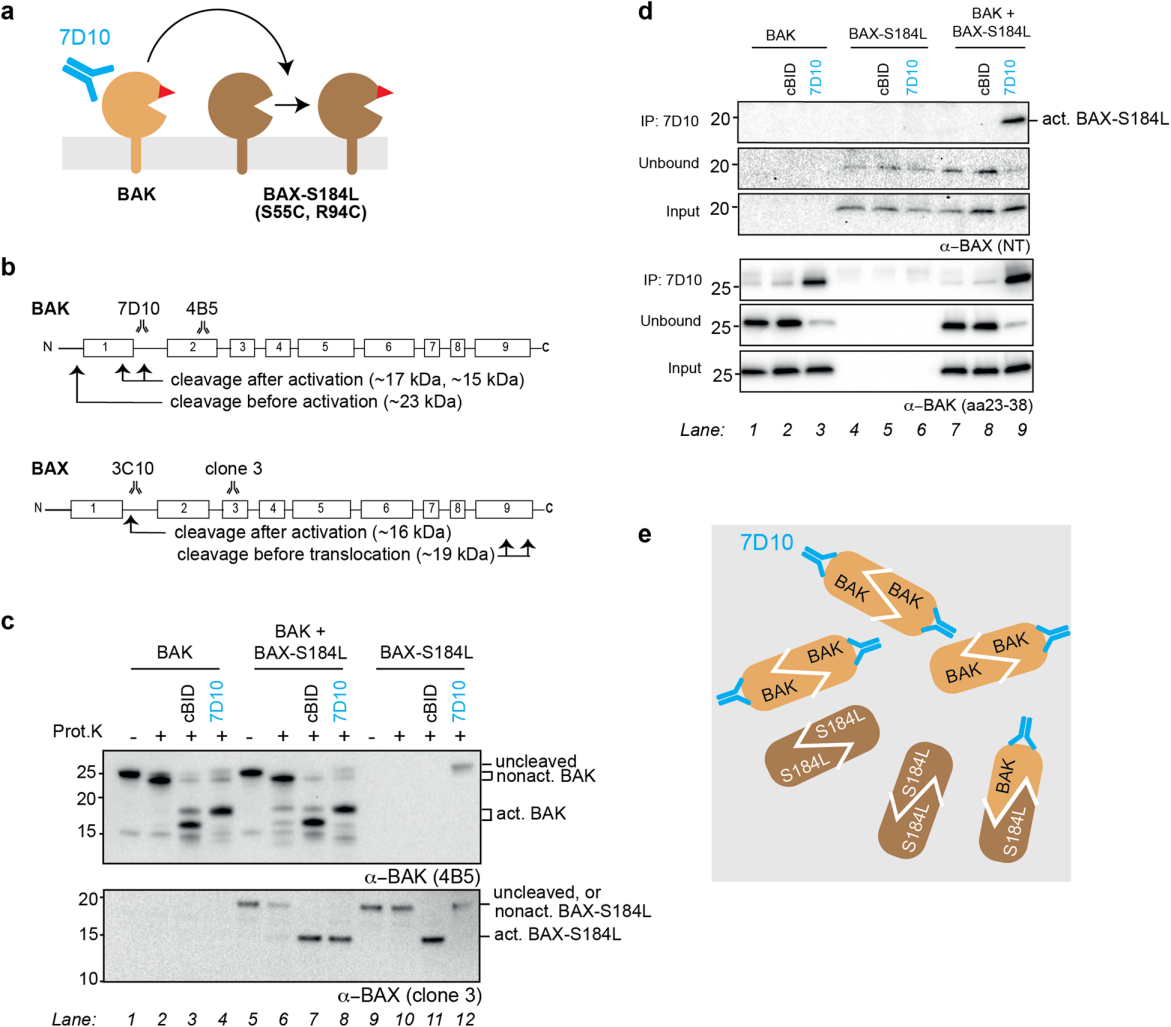
Antibody-activated BAK can activate mitochondrial BAX. **a** Schematic of BAK co-expressed with a mitochondrial form of BAX (BAX-S184L) and incubated with the 7D10 antibody to activate BAK. **b** Major proteinase K cleavage sites in activated BAK and BAX locate to the α1–α2 loop region. Fragment sizes detected by the 4B5 or clone 3 antibody (see Fig. 3c) are shown. Note that activated BAK is cleaved to a 17 kDa fragment (rather than a 15 kDa fragment) when 7D10 antibody is bound, because the antibody masks a cleavage site. **c** Conformation change in BAX-S184L is triggered by 7D10 antibody if BAK is also present. BAK and BAX-S184L were expressed individually or together in *Bax*^*−/−*^*Bak*^*−/−*^ MEF, and membrane fractions incubated with the 7D10 antibody to activate BAK. As positive controls, aliquots were also incubated with cBID to activate both BAK and BAX-S184L. Samples were then incubated with proteinase K and western blotted to reveal fragments corresponding to nonactivated and activated forms of BAK (upper panel) or BAX-S184L (lower panel). Data are representative of two independent experiments. **d** Co-immunoprecipitation shows that BAX-S184L is activated by 7D10 antibody if BAK is also present. Membrane fractions were treated as in (**c**) and immunoprecipitated with 7D10, which had been added to the incubations to activate BAK. The samples were then immunoblotted for BAX-S184L (upper panels) or BAK (lower panels). Data are representative of two independent experiments. **e** Schematic of BAK and BAX-S184L dimers captured by the 7D10 antibody in panel (**d**). Activated BAK and activated BAX-S184L associate to form dimers, some of which 7D10 can immunoprecipitate.

To test whether BAK can activate BAX when both are located on the MOM, we co-expressed BAK and mitochondria-targeted BAX-S184L (with cysteine substitutions in the BH3 domain (S55C) and groove (R94C)), and incubated the membrane fractions with 7D10 antibody to directly activate BAK (Fig. 3a).

We first assessed activation of BAK and BAX-S184L using limited proteolysis by proteinase K. As illustrated in Fig. 3b, it cleaves nonactivated BAK and BAX at their far N- and C-termini, respectively, but their activation exposes cleavage sites in the α1–α2 loop, and the cleaved fragments can be identified by the relevant antibodies, 4B5 for BAK and clone 3 for BAX^45^. As expected, cBID added to the membrane fractions activated both BAK and BAX-S184L, whether expressed individually or together (Fig. 3c, lanes 3, 7, and 11). In addition, the 7D10 antibody activated BAK but not BAX-S184L when individually expressed (Fig. 3c, lanes 4 and 12). However, when BAK and BAX-S184L were co-expressed, 7D10 also activated BAX-S184L to an extent similar to cBID (Fig. 3c, lower panel, lane 8 vs. 7). Thus, 7D10-activated BAK efficiently activated full-length BAX anchored in the mitochondrial outer membrane.

We next demonstrated activation of BAX-S184L by its ability to form oligomers, as assessed by co-immunoprecipitation (Fig. 3d) or linkage (Fig. S3). BAX-S184L immunoprecipitated with the 7D10 antibody if and only if BAK was present (Fig. 3d, upper panels, lane 9 vs. 3 and 6), indicating that a portion of BAX-S184L had become activated and could dimerize with activated BAK (Fig. 3e). In addition, a large portion of BAX-S184L formed homodimers following 7D10 treatment, as shown by disulfide linkage between cysteines in its BH3 domain and groove (S55C:R94C reciprocal linkage), but again only when BAK was present (Figure S3, upper panel, lane 12 vs. 8).

In summary, three methods of detecting activated BAX-S184L (limited proteolysis, co-immunoprecipitation, cysteine linkage) showed that 7D10-activated BAK could activate this mitochondrial form of BAX, and that its activation was near-complete. Furthermore, because BH3:groove dimers formed between the activated BAX-S184L molecules (Fig. S3), autoactivation can be a transient or “hit-and-run” process, similar to activation by BH3-only proteins such as BID and BIM, which dissociate from the activated monomer.

### Antibody-activated BAK at mitochondria recruits and activates wild-type BAX

**Fig. 4:**
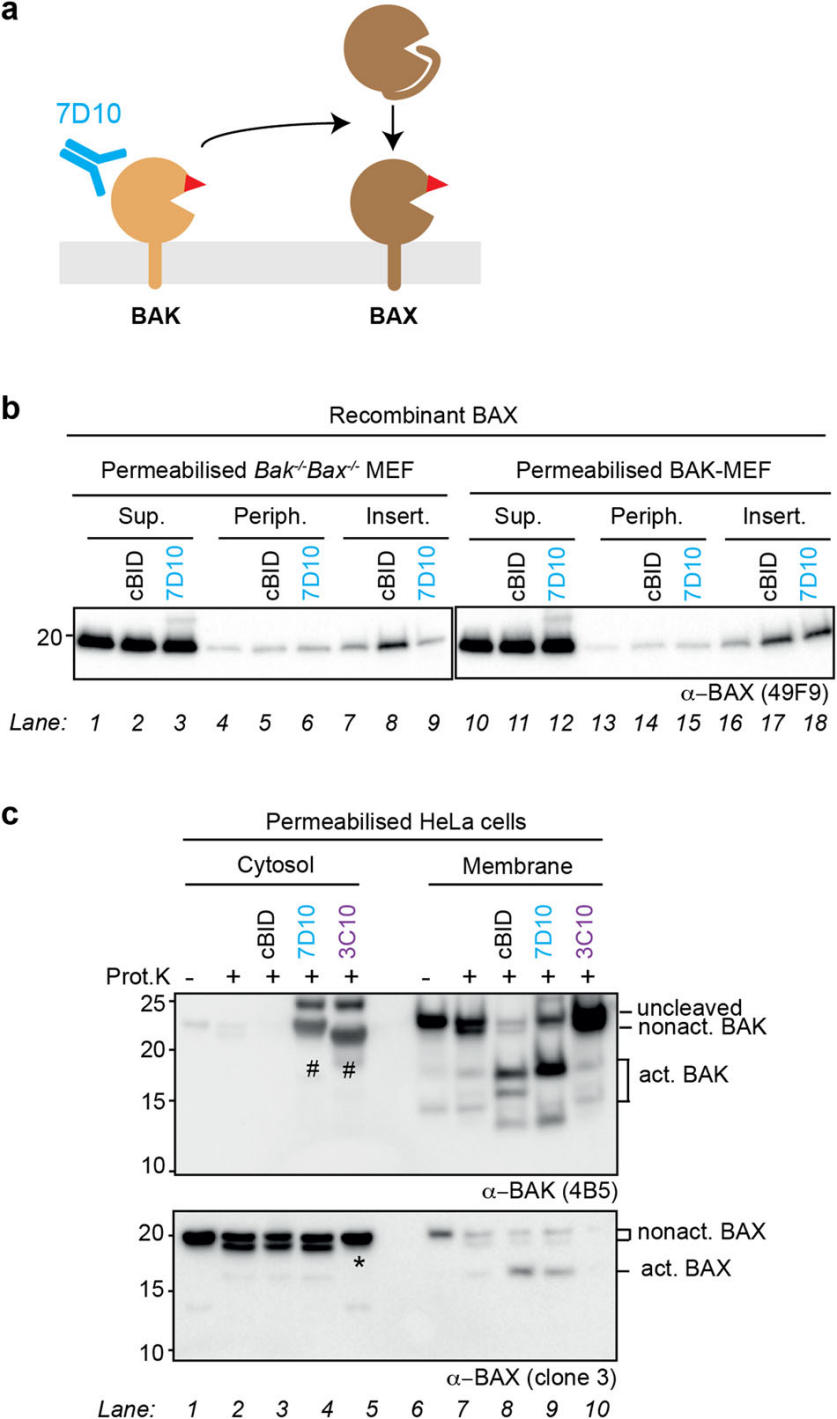
Antibody-activated BAK at mitochondria recruits and activates wild-type BAX. **a** Schematic of BAK combined with wild-type BAX and incubated with the 7D10 antibody to activate BAK. **b** Translocation and MOM insertion of recombinant BAX is triggered by 7D10 if BAK is also present. *Bax*^*−/−*^*Bak*^*−/−*^ MEF or those cells expressing BAK were permeabilized and supplemented with 20 nM recombinant BAX, then incubated with 7D10 to activate BAK. As positive controls, aliquots were also incubated with cBID to activate both BAK and BAX. Samples were then centrifuged to recover the supernatant (Sup). The pellet fraction was then subjected to carbonate extraction to separate peripherally attached (Periph) and membrane-inserted (Insert) BAX. The three fractions were then immunoblotted for BAX. Data are representative of three independent experiments. **c** Translocation and activation of cytosolic BAX in permeabilized HeLa cells incubated with 7D10. HeLa cells were permeabilized and incubated with cBID, the 7D10 antibody to BAK, or the 3C10 antibody to BAX. The cytosolic and membrane fractions were then incubated with proteinase K and blotted for BAK (upper panel) or BAX (lower panel). Data are representative of two independent experiments. *Inhibitory effect of 3C10 on cytosolic BAX^44^ is evident from the inability of proteinase K to cleave α9. ^#^ Cross-reactive fragments from 7D10 and 3C10 antibodies.

To determine if BAK could also activate cytosolic wild-type BAX, we combined BAX with mitochondria that contained BAK and incubated the mixture with 7D10 (Fig. 4a). In one approach, recombinant BAX was incubated with permeabilized MEF, either *Bak*^*−/−*^*Bax*^*−/−*^ MEF or those cells expressing human BAK (Fig. 4b). The samples were then centrifuged, and the pelleted membrane fractions subjected to carbonate extraction to separate peripherally attached from membrane-inserted proteins. As expected, cBID elicited BAX translocation and insertion into the mitochondria-enriched membrane fraction, in the presence or absence of BAK (Fig. 4b, lanes 8 and 17). BAX translocation and MOM insertion also followed 7D10 treatment, but only if BAK was present (Fig. 4b, lane 18 vs. lane 9). Thus, antibody-activated BAK can recruit BAX to insert into the MOM, and at an efficiency comparable to cBID.

A second approach used HeLa cells to test activation between endogenous BAK and BAX proteins. Permeabilized HeLa cells were incubated with cBID, 7D10, or 3C10, and the cytosol and membrane fractions separated and assessed for BAK and BAX activation (Fig. 4c). As expected, either cBID or 7D10 activated BAK, as indicated by limited proteolysis (Fig. 4c, upper panel, lanes 8 and 9). Notably, either cBID or 7D10 also promoted BAX activation, as shown by the proteinase K-cleaved BAX in the membrane fraction (Fig. 4c, lower panel, lanes 8 and 9). As a negative control, the anti-BAX antibody 3C10 did not trigger BAX translocation or activation (Fig. 4c, lower panel, lane 10), and decreased proteinase K cleavage of BAX α9 (Fig. 4c, lower panel, lane 5), as we reported previously^43,44^. In summary, 7D10-activated BAK can activate the BAX present in HeLa cytosol, and to an extent equivalent to cBID.

### Antibody-activated mitochondrial BAX-S184L does not activate BAK

As antibody-activated BAK could activate both BAK and BAX in a variety of contexts, we next tested if antibody-activated BAX also had this ability. This seemed likely as BAX BH3 peptides and BID^BAXBH3^ chimeras strongly activated both BAK and BAX in mitochondrial experiments^29,36^.

**Fig. 5:**
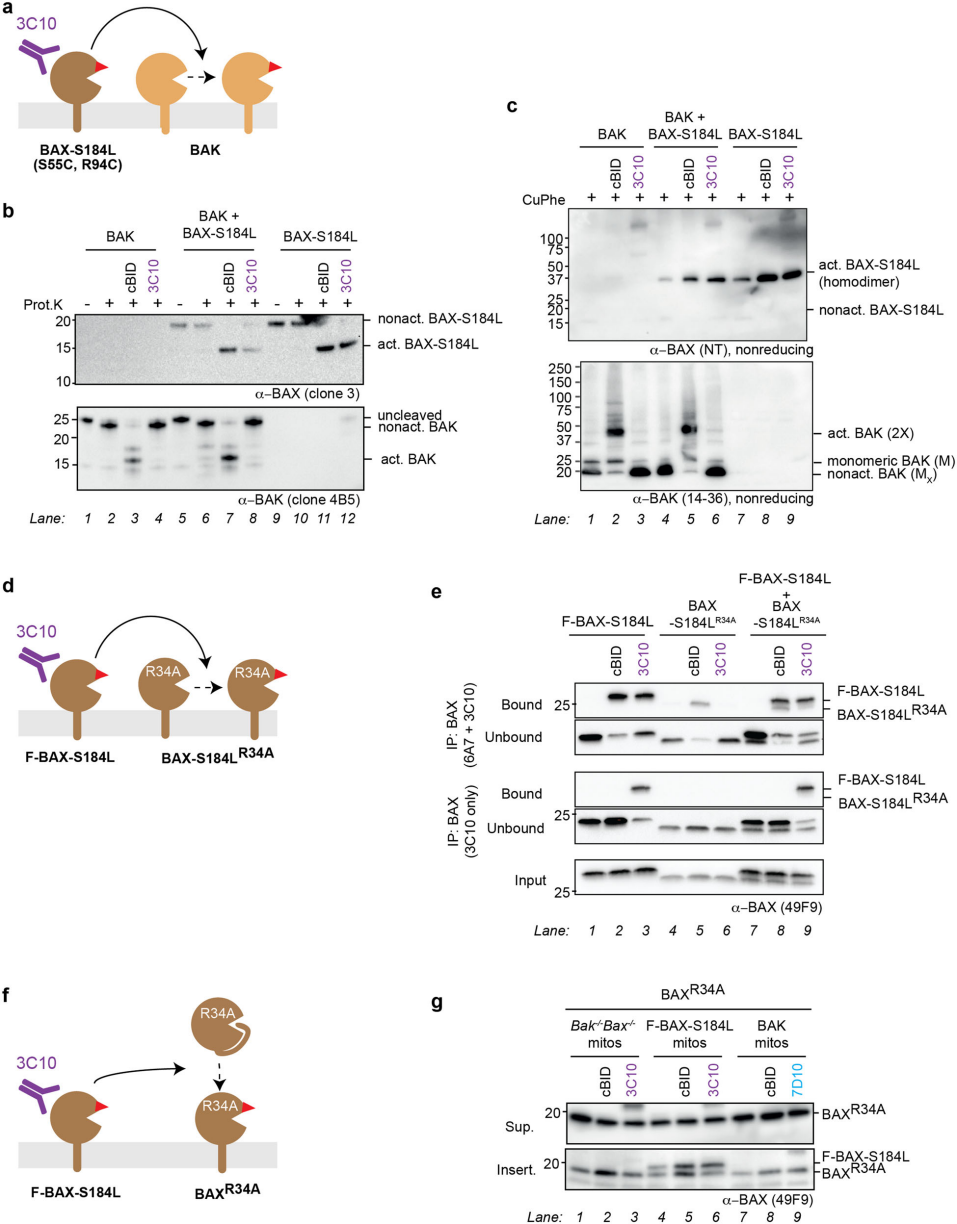
Antibody-activated mitochondrial BAX-S184L is a poor activator of BAK, BAX-S184L, and BAX. **a** Schematic of BAX-S184L co-expressed with BAK and incubated with 3C10 antibody, which directly activates BAX-S184L. **b** Conformation change in BAK is not triggered by 3C10 antibody, even if BAX-S184L is activated. BAX-S184L and BAK were expressed individually or together in *Bax*^−*/−*^*Bak*^*−/−*^ MEF, and membrane fractions incubated with the 3C10 antibody to activate BAX-S184L. As positive controls, aliquots were also incubated with cBID to activate both BAX-S184L and BAK. Samples were then incubated with proteinase K and western blotted to reveal fragments corresponding to nonactivated and activated forms of BAX-S184L (upper panel) or BAK (lower panel). Data are representative of two independent experiments. **c** Oligomerization of BAK is not triggered by 3C10 antibody, even if BAX-S184L has been activated and oligomerized. Aliquots from (**b**) were incubated with oxidant (CuPhe) to detect oligomerization. Note that BAX-S184L linkage at S55C:R94C detects BH3:groove homodimers (see schematic in Fig. S3), and that BAK linkage detects both C14:C166 linkage within nonactivated BAK (Mx) and linkage within and between dimers (2X). Data are representative of two independent experiments. **d** Schematic of F-BAX-S184L co-expressed with the BAX-S184L^R34A^ variant and then incubated with 3C10 which directly activates BAX-S184L but not the R34A variant. **e** Immunoprecipitation shows that BAX-S184L is a poor activator of BAX-S184L^R34A^. BAX-S184L and BAX-S184L^R34A^ were expressed individually or together in *Bax*^*−/−*^*Bak*^−*/*−^ MEF, and membrane fractions incubated with the 3C10 antibody to activate BAX-S184L. As positive controls, aliquots were also incubated with cBID to activate both BAX-S184L variants. The pellet fractions were then solubilized and an aliquot taken for the input sample. Additional aliquots were immunoprecipitated either with (upper panels) or without (lower panels) addition of the 6A7 antibody that binds to activated BAX, and the samples immunoblotted for BAX. Note that the beads used in the immunoprecipitation would bind both the 3C10 and 6A7 antibodies. Data are representative of two independent experiments. **f** Schematic of BAX-S184L combined with recombinant BAX^R34A^ and incubated with the 3C10 antibody to directly activate BAX-S184L. 3C10 cannot bind the BAX^R34A^ variant. **g** BAX-S184L is a poor activator of recombinant BAX^R34A^. Membrane fractions from *Bax*^−*/−*^*Bak*^−*/−*^ MEF or those cells expressing BAX-S184L or BAK were supplemented with 50 nM recombinant BAX^R34A^ and incubated with cBID or the 3C10 or 7D10 antibody. Samples were then subjected to carbonate extraction as in Fig. 4b and the supernatant (Sup.) and inserted (Insert.) fractions blotted for BAX. Note that F-BAX-S184L is partly peripherally attached, but becomes activated and inserted following either cBID or 3C10 treatment (lanes 5 and 6). Data are representative of three independent experiments.

We first explored if mitochondria-targeted BAX-S184L activated by the 3C10 antibody could activate BAK (Fig. 5a). Limited proteolysis indicated that 3C10 could activate BAX-S184L as expected (Fig. 5b, upper panel, lanes 8 and 12), but BAK remained in its nonactivated form (Fig. 5b, lower panel, lanes 4 and 8). When assessed by oxidant-induced cysteine linkage, 3C10 produced BAX-S184L homodimers as expected (Fig. 5c, upper panel, lanes 6 and 9). However, no activation of BAK was evident, as indicated by the absence of 2× complexes and the presence of the nonactivated BAK M_x_ band (intramolecular cysteine linkage that is a hallmark of nonactivated BAK) (Fig. 5c, lower panel, lane 6). Thus, full-length mitochondrial BAX-S184L is a poor activator of BAK.

### Mitochondrial BAX-S184L is a poor activator of mitochondrial BAX-S184L^R34A^

To test for activation between BAX molecules residing in the MOM, we exploited the R34A mutation, which abrogates binding of 3C10 to BAX^44^. We paired F-BAX-S184L with BAX-S184L^R34A^ and directly activated the former with the 3C10 antibody (Fig. 5d). One set of samples was then immunoprecipitated for the 3C10 antibody that had been added as the stimulus, while another set of samples was supplemented with the 6A7 antibody to immunoprecipitate all forms of activated BAX-S184L. As expected, the addition of 6A7 allowed immunoprecipitation of both BAX-S184L variants from the cBID incubations (Fig. 5e, upper panels, lanes 2, 5, 8 vs. 1, 4, and 7). However, 6A7 did not allow immunoprecipitation of the R34A variant from 3C10-treated samples (Fig. 5e, upper panels, lanes 6 and 9). Thus, even if F-BAX-S184L was present and able to be activated by 3C10, it was a poor activator of BAX-S184L^R34A^.

### Mitochondrial BAX-S184L is a poor activator of cytosolic BAX^R34A^

To test for activation of cytosolic BAX by mitochondrial BAX-S184L, we paired F-BAX-S184L with recombinant BAX^R34A^ and again directly activated the former with the 3C10 antibody (Fig. 5f). To provide relevant controls, incubations included membrane fractions from three types of cells: *Bak*^*−/−*^*Bax*^*−/−*^ MEF and those cells re-expressing either F-BAX-S184L or BAK. Recombinant BAX^R34A^ was added to all incubations and direct activation of F-BAX-S184L or BAK initiated with cBID, 3C10, or 7D10 (Fig. 5g). Samples underwent carbonate extraction to identify BAX that had inserted into the MOM. As expected, cBID triggered BAX^R34A^ to translocate and insert into mitochondria (Fig. 5g, lower panel, lanes 2, 5, and 8 vs. 1, 4, and 7). In addition, the 7D10 antibody triggered insertion of BAX^R34A^ in the presence of BAK (Fig. 5e, lower panel, lane 9 vs. 7), confirming the ability of BAK to activate cytosolic BAX (Fig. 4b). Notably, while the 3C10 antibody increased insertion of F-BAX-S184L, some of which may be peripherally attached, increased insertion of BAX^R34A^ was not evident (Fig. 5g, lower panel, lane 6 vs. 4). Thus, even if F-BAX-S184L was activated by 3C10, it was a poor activator of cytosolic BAX.

### Mutation in its hydrophobic groove converts BAX into a strong activator

**Fig. 6:**
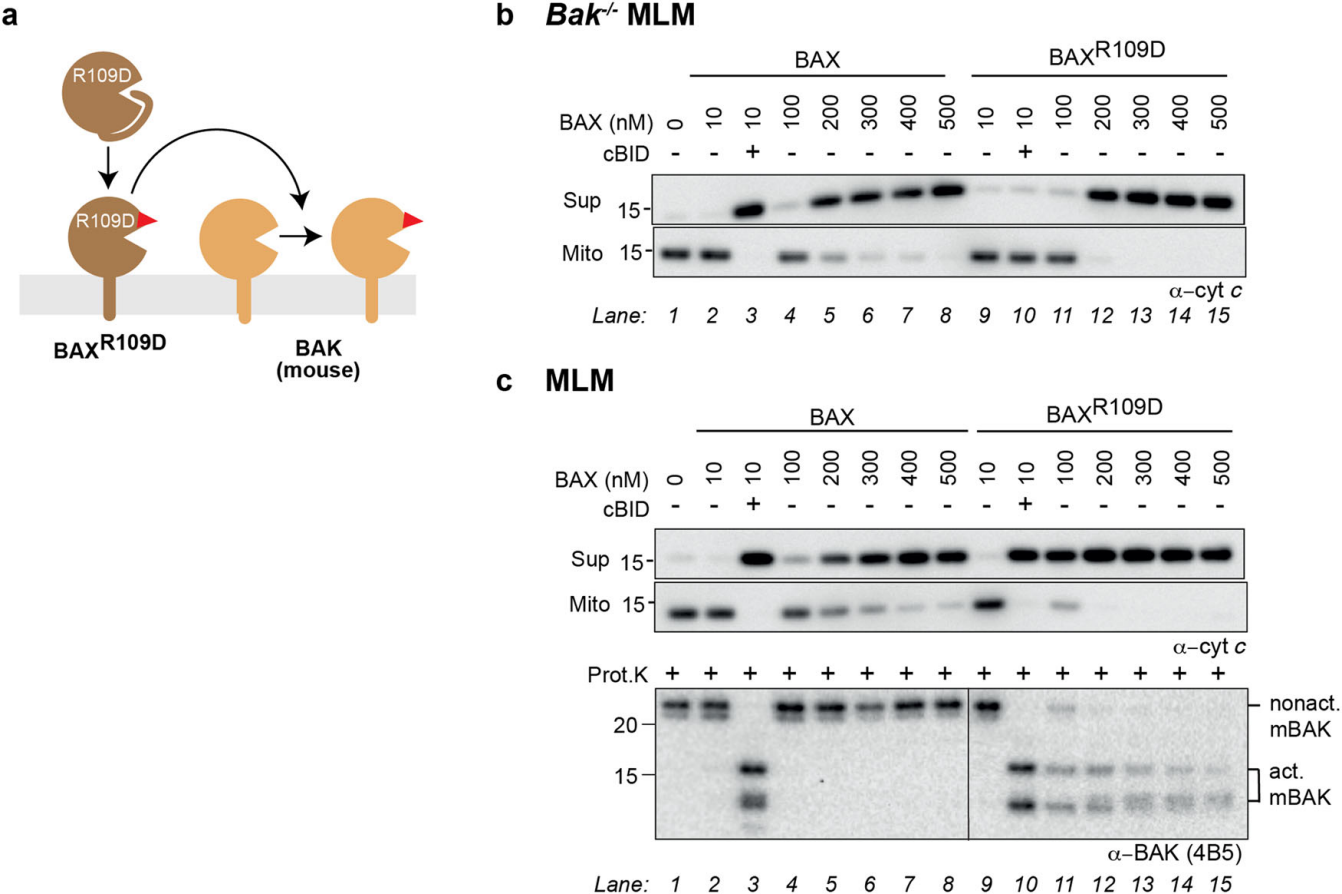
Mutation in hydrophobic groove converts BAX into a strong activator. **a** Schematic of recombinant BAX^R109D^ incubated with BAK-expressing mouse liver mitochondria. **b** In the absence of an activating stimulus (e.g., no cBID or activating antibody), intermediate levels of BAX or BAX^R109D^ can permeabilize *Bak*^*−/−*^ mitochondria. Mouse liver mitochondria (MLM) from *Bak*^*−/−*^ mice were incubated with increasing levels of recombinant BAX or the groove mutant BAX^R109D^ and assessed for cytochrome *c* release. As positive controls, additional aliquots were incubated with 10 nM cBID and 10 nM BAX proteins. Data are representative of four independent experiments. **c** Activated BAX^R109D^ but not wild-type BAX is able to autoactivate BAK. MLM from wild-type mice were incubated as in (**b**) and assessed for cytochrome *c* release (upper panels) and activation of mouse BAK (by proteinase K cleavage, lower panels). Data are representative of four independent experiments.

We next considered whether poor autoactivation by full-length activated mitochondrial BAX might be due to incomplete exposure of the BH3 domain, or to an inability to access the activation sites in nonactivated BAK or BAX. To explore this, we exploited the ability of intermediate levels of BAX to permeabilize mitochondria even in the absence of an apoptotic stimulus such as cBID. Others have shown that in cells deficient for all BCL-2 family proteins, BAX expression alone was sufficient to induce apoptosis^48^. To enhance the likelihood of BAX BH3 exposure, we also tested the groove mutant BAX^R109D^ (Fig. 6a), which has a low affinity for cBID^12,38^. We reasoned the low affinity for BH3 domains may also reduce BH3:groove homodimer formation.

Before testing for BAX autoactivation of BAK, we assessed the intrinsic pore-forming activity of the two BAX variants in *Bak*^*−/*−^ MLM (Fig. 6b). As previously observed^38^, in response to cBID, low levels of BAX but not BAX^R109D^ released cytochrome *c* from *Bak*^*−/−*^ MLM (Fig. 6b, lane 3 vs. 10). In the absence of cBID, however, 200 nM of either BAX variant released cytochrome *c* (Fig. 6b, lanes 5 and 12). Moreover, pore formation by this concentration of BAX was BH3-dependent, as an antibody (10F4) to the BAX BH3 region (e.g., α2–α3 hinge, residues 66–78) blocked cytochrome *c* release (Fig. S4a–d). We previously reported similar inhibition of BAK-induced cytochrome *c* release by an antibody (4B5) to the BAK α2–α3 hinge region^17^. Notably, 10F4 did not inhibit BAX or BAX^R109D^ translocation (Fig. S4e), indicating that translocation is not due to BAX BH3 exposure in a portion of the recombinant BAX proteins. These experiments in *Bak*^*−/*−^ MLM establish that intermediate levels of either BAX or BAX^R109D^ induce cytochrome *c* release in a BH3-dependent manner.

Since both BAX variants exposed their BH3 domain under these conditions, we next tested their ability to activate BAK by incubating each with MLM, which contains mouse BAK (mBAK; Fig. 6a). Strikingly, mBAK was activated by 100 nM (or higher) BAX^R109D^ (Fig. 6c, lower panel, lanes 11–15) and contributed to cytochrome *c* release (compare lane 11 in Fig. 6b and c, upper panel). In contrast, mBAK was not activated by even 500 nM of wild-type BAX alone (Fig. 6c, lower panel, lanes 4–8). Autoactivation by BAX^R109D^ required its BH3 domain, as the 10F4 antibody prevented mBAK activation (Fig. S4d, lower panels, lanes 20–22 vs. 9–11). Thus, the groove mutation allowed full-length activated BAX to activate BAK, which promoted release of cytochrome *c*.

In summary, in the absence of cBID, intermediate concentrations of full-length BAX or BAX^R109D^ could translocate to the mitochondrial fraction and expose the α2–α3 region as a step toward cytochrome *c* release. Exposure of the α2–α3 region in BAX^R109D^ (but not wild-type BAX) provoked BAK activation, indicating that the BH3 domain in the activated BAX^R109D^ monomer can access the activation site in mBAK.

## Discussion

The concept of autoactivation arose from the ability of truncated versions of BAK and BAX to activate full-length BAK and BAX^33^. To investigate whether autoactivation can occur between full-length proteins located on the MOM, we co-expressed or co-incubated pairs of BAK and BAX variants and specifically activated one of the variants with an antibody, then tested whether that variant could activate the paired protein. We found that full-length BAK was a strong activator of each partner (Figs. 1–4). Surprisingly, despite the very similar structure and function of BAK and BAX in most analyses, both full-length BAX and BAXS184L proved weak activators of their partners (Figs. 5 and 6).

The robust ability of BAK but not BAX to activate other effector molecules suggests a significant difference between their exposed BH3 domains in the context of the full-length proteins. As peptides, and even in the context of chimeric BID proteins, the BAX BH3 sequence seems as potent as the BAK BH3 in activating BAK or BAX^29,36^, with some exceptions^13^. The inactivity of BAX is not explained by the exposed BH3 domain adopting the incorrect orientation for binding to other BAK or BAX molecules, as BAX^R109D^ could autoactivate mouse BAK in a BH3-dependent manner (Figs. 6 and S4). One possibility is that the half-life of the activated BAX monomer is very short compared to that of the activated BAX^R109D^ monomer, due to the R109D groove mutation slowing conversion to symmetric homodimers. Activated BAK may also be slow to dimerize, at least compared to wild-type BAX. The basis for slow dimerization of BAK but not BAX is unclear because the BAK and BAX α2–α5 core domain homodimers show similar shape complementarity and buried surface area (personal communication, Peter Colman). However, other factors such as initial binding affinities or lateral mobility in the MOM may differ for the activated BAK and BAX monomers.

While our experiments are largely based on using antibodies rather than physiological stimuli (e.g., BH3-only proteins) to trigger initial activation of BAK or BAX, our findings may also hold for BH3-only signaling. One consideration was that the extra bulk of the 7D10 antibody bound to the flexible N-terminus may slow BAK homodimerization and increase the window in which the monomer can act as an activator. However, an attached antibody might be expected to affect BAK and BAX similarly, as the structures of BAK homodimers and BAX homodimers are similar^12,13^. In addition, BAK activated by the small 7D10 single-chain variable fragment (~25 kD) rather than the full IgG antibody (~150 kD) was also a strong autoactivator (Fig. S5). Finally, even without antibody-activation, wild-type BAX was a poor activator (Figs. 6 and S4).

The activation site(s) on BAK, BAX-S184L, and wild-type BAX (Fig. S1b) must be accessible to the exposed BH3 domain of BAK. For BAK activation of cytosolic BAX, BAX must locate to the MOM. This BAX might correspond to the population that is peripherally attached to the MOM^49^, or that associates with VDAC2^50^, or that retrotranslocates to the cytosol^51–53^. Cytosolic BAX may also collide with mitochondria without requiring a translocation stimulus^29^. Interestingly, BAK activation of BAX may contribute significantly to BAX translocation and activation (Figs. 4 and 5g), consistent with recent genetic evidence that either BAK or VDAC2 can promote BAX translocation and activation^54^. However, contrary to previous studies^32,34^, our data provide little evidence that BAX directly contributes to BAX translocation or activation.

**Fig. 7:**
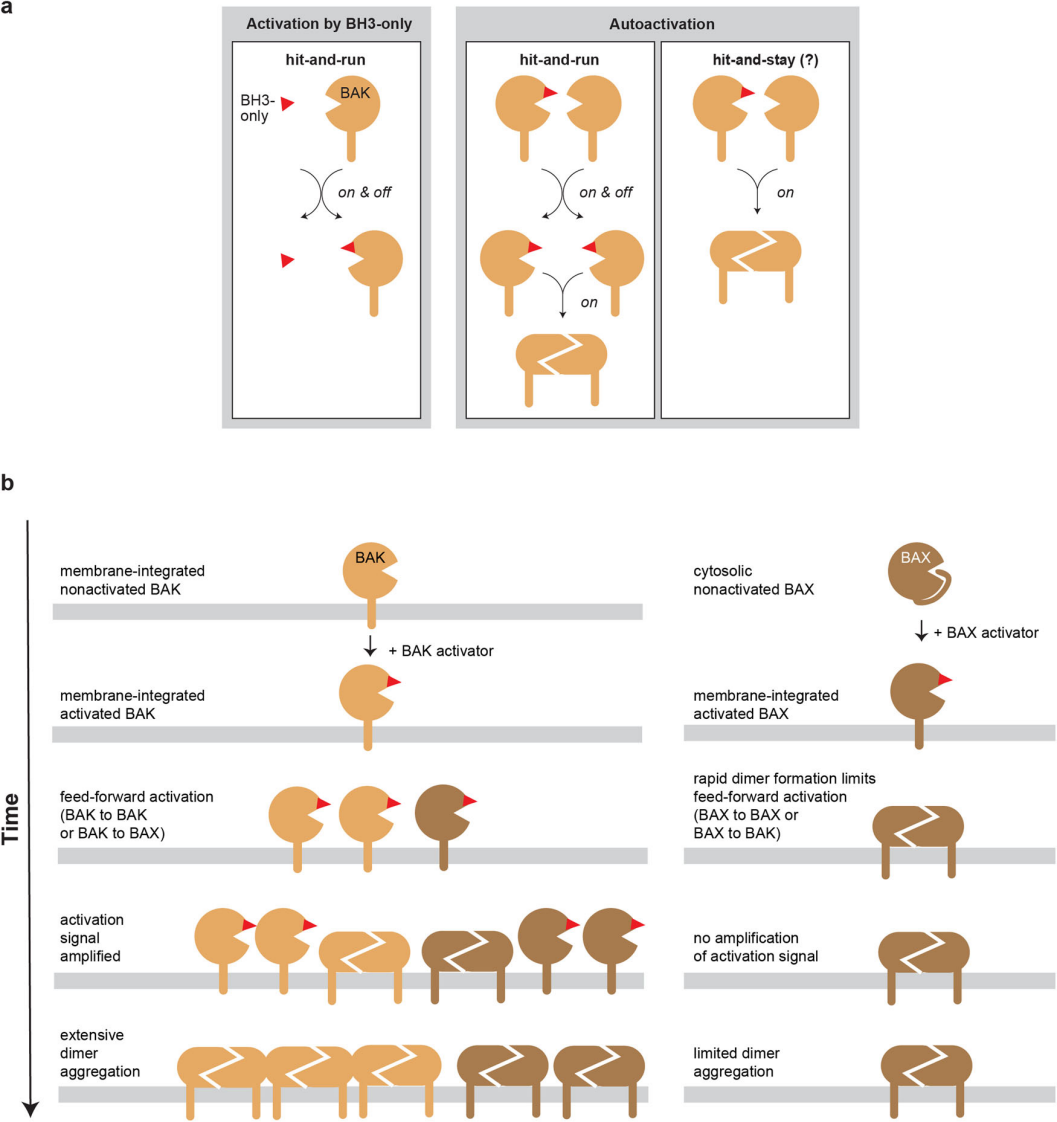
Summary. **a** Schematic of the transient interaction involved in autoactivation by BAK. The α2–α5 hydrophobic groove activation site in BAK is used to illustrate the hit-and-run interaction involved in its activation by BH3-only proteins (left panel). Autoactivation also involves a transient interaction (middle panel) as the targeted BAK^G51C^ and BAX-S184L proteins became nearly fully activated and could homodimerize rather than remain bound to BAK (see Figs. S2, 3c, and S3). Whether a proportion of targeted molecules can remain bound to BAK as a BH3:groove dimer (right panel; hit-and-stay) has not been determined. **b** The transient interaction involved in autoactivation by BAK (left panels) highlights the potential of directly targeting BAK to either inhibit or promote apoptosis, whereas direct BAX activation (right panels) is expected to be less productive.

Our data also address whether autoactivation involves a transient interaction (hit-and-run) or a stable reciprocal BH3:groove interaction (hit-and-stay) (Fig. 7a). For example, because both activated BAK and its targeted BAK or BAX molecules possess a hydrophobic groove, an initial unidirectional BH3:groove interaction may progress to reciprocal BH3:groove interactions, to generate a dimer that cannot itself be an activator. Our data indicate, however, that the activating interaction is often transient, as 7D10-activated BAK activated most of the co-expressed BAK^G51C^ (Fig. S2a, b) and BAX-S184L (Fig. S3b) proteins. Furthermore, a large proportion of the autoactivated proteins did not remain bound to BAK. For example, only a proportion of BAK^G51C^ molecules co-precipitated with 7D10-activated BAK (Fig. 2b, lane 9) despite all BAK^G51C^ being activated (Fig. S2a, b). Furthermore, most BAX-S184L formed homodimers that could be linked via a BH3:groove interaction within those dimers (Fig. S3). The transient interaction involved in autoactivation may be explained just as for activation by BH3-only proteins^12,14,37,55,56^: binding induces major conformation change in the α2–α5 activation site (or α1/6 pocket) to decrease affinity for the activator (see animation in Fig. S6).

The transient nature of BAK autoactivation implies a catalytic process that may be important to recruit sufficient BAK and BAX into homodimers to ensure apoptotic death (Fig. 7b). If signaling by BH3-only proteins is limiting, any BAK that became activated could augment that signal to activate more BAK (and BAX) to push the cell over the threshold of BAK and BAX homodimerization required for pore formation. Notably, BAK is not expressed in differentiated neurons^57–59^. Its silence there may have evolved to protect the neuronal system against accidental apoptosis, by removing both BAK’s intrinsic pore-forming capacity and the feed-forward nature of autoactivation.

The prosurvival BCL-2 proteins, especially MCL-1 and BCL-X_L_, can sequester activated BAK, an inhibition process referred to as Mode 2^27–29,60–62^ (see Fig. 1). Sequestration involves binding of the exposed BAK BH3 domain, which would inhibit not only homodimer formation, but autoactivation, as argued previously^31,34,48,63^. Thus, in cancer cells that resist treatment due to increased prosurvival proteins, the proteins may act in part via preventing autoactivation by BAK. Accordingly, inhibitors of prosurvival proteins, in particular BH3 mimetics that target MCL-1 and BCL-X_L_ that are currently in clinical trials^64^, may act in part by promoting autoactivation by BAK.

Several means of directly regulating BAK and BAX have been reported, as reviewed in ref. ^2^. Autoactivation by BAK implies that activators or inhibitors of BAK rather than of BAX, may be important therapeutic agents. Direct activators of BAK may best recruit additional pore-forming BAK and BAX molecules to induce robust apoptosis in cancer cells. Direct inhibitors of BAK, including small molecules^65^, by precluding autoactivation, may be particularly effective in inhibiting unwanted apoptosis.

## Supplementary information

Supplementary Figure and Table Legends

Supplementary Figure 1

Supplementary Figure 2

Supplementary Figure 3

Supplementary Figure 4

Supplementary Figure 5

Supplementary Video - Figure S6

Supplementary Table 1
