# Supplementary material for: Robust autoactivation for apoptosis by BAK but not BAX highlights BAK as an important therapeutic target
Source: Cell Death Dis. 2020 Apr 23;11(4):268. doi: 10.1038/s41419-020-2463-7 (PMC7181796; doi:10.1038/s41419-020-2463-7)
Supplement: Supplementary file 1 — Supplementary Figure and Table Legends [file 41419_2020_2463_MOESM1_ESM.docx]

**Supplementary Figure 1. Structural features of BAK and BAX involved in autoactivation**

**a.** Sequence alignment of BH3 domains found to activate BAK and BAX in mitochondria experiments^36^. Indicated are the signature hydrophobic h1-4 residues, which together with the conserved aspartate residue (underlined), are important for binding to prosurvival proteins^66^ and to BAX^12^.

**b.** Activation sites in BAX. A model of BAX anchored to the mitochondrial outer membrane via the C-terminal α9 helix shows that the α8-α9 linker may allow all three proposed activation sites to be accessed by activators. Schematic generated in PyMol using PDB files 1F16, 4BD2 and 2K7W. Sidechains of BH3 domain h1-4 and (red) and 3C10 epitope at the α1-α2 loop N-terminus (white) are shown.

**Supplementary Figure 2. Antibody-activated BAK activates BAK^G51C^**

**a.** Immunoprecipitation shows that F-BAK^G51C^ is activated by 7D10 antibody if BAK is also present. As in Figure 2, two BAK variants were expressed individually or together in *Bax^-/-^Bak^-/-^* mouse embryonic fibroblasts (MEF), and membrane fractions incubated with cBID, or with the 7D10 antibody. For immunoprecipitation, a mouse antibody (14-36) that recognizes activated BAK was added, and samples immunoblotted for BAK (Ab-1, upper panel) or for the FLAG epitope present in F-BAK^G51C^ only (lower panel). Schematic (right) illustrates that the beads used in the immunoprecipitation would bind the 7D10 antibody added to activate BAK, as well as the 14-36 antibody. Data are representative of two independent experiments.

**b.** Oligomerization of F-BAK^G51C^ is triggered by 7D10 antibody if BAK is also present. Membrane fractions from (a) were incubated with an oxidant (CuPhe) to induce disulfide bonds between activated BAK proteins, then run on nonreducing SDS-PAGE and probed for BAK. Note that linkage results in 2X complexes of three sizes, as illustrated by disulfide linkage (S-S) of cysteine residues present in the two BAK variants (right), and as indicated by our previous linkage studies ^1^. Data are representative of two independent experiments.

**Supplementary Figure 3. Antibody-activated BAK can activate mitochondrial BAX**

Homodimers of BAX-S184L are generated by incubation with 7D10 antibody if BAK is also present. As in Figure 3, BAK and BAX-S184L were expressed individually or together in *Bax^-/-^Bak^-/-^* MEF and membrane fractions incubated with 7D10 to activate BAK. Samples were then incubated with the oxidant (CuPhe) to induce disulfide bonds in the activated BAK and BAX-S184L proteins. Samples were run on nonreducing SDS-PAGE and probed for BAX (upper panel) or BAK (lower panel). Schematic (right) shows how linkage of cysteine residues may generate linked complexes. Note that the BAX-S184L cysteine residues in the BH3 domain (S55C) and groove (R94C) exclusively link BH3:groove homodimers of BAX-S184L (i.e. no linkage between dimers). Data are representative of two independent experiments.

**Supplementary Figure 4. Antibody to the BAX α2-α3 hinge region blocks cytochrome *c* release by BAX**

**a.** The 10F4 antibody maps to the α2-α3 hinge region of BAX. A rat monoclonal antibody raised against the BAX 66-78 peptide (green) was tested for binding to BAX peptides by peptide array (left panel), performed as previously^11^. Error bars represent the SD of the mean of at least three independent experiments.

**b.** The 10F4 epitope maps to BAX α2-α3 corner including part of the BH3 domain. Schematic of the BAX α2-α5 core dimer was generated in PyMol (PDB:4BDU). Sidechains of BH3 domain h1-4 and (red) are shown.

**c.** The 10F4 antibody blocks cytochrome *c* release mediated by intermediate levels of BAX or BAX^R109D^. Mouse liver mitochondria (MLM) from *Bak^-/-^* mice were incubated with increasing levels of recombinant BAX or the groove mutant BAX^R109D^ and assessed for cytochrome *c* release. As positive controls, aliquots were also incubated with 10 nM cBID and 10 nM BAX proteins. Note that addition of the 10F4 antibody at the start of the incubation (right) prevented cytochrome *c* release in all samples except for those containing cBID-activated BAK. Data are representative of two independent experiments.

**d.** The 10F4 antibody blocks the ability of BAX^R109D^ to activate BAK. MLM from wild-type mice were incubated as in panel c and assessed for cytochrome *c* release (upper panels) and activation of mouse BAK (by proteinase K cleavage, lower panels). Note that addition of the 10F4 antibody at the start of the incubation (right) prevented activation of mouse BAK in all samples except for those containing cBID. Data are representative of two independent experiments.

**e.** Translocation of BAX or BAX^R109D^ to the mitochondrial fraction is independent of BH3 exposure. Mitochondrial fractions from panels c and d were immunoblotted for BAX. Data are representative of two independent experiments.

**Supplementary Figure 5. BAK activated by an antibody fragment is also a strong autoactivator**

BAK was expressed in *Bax^-/-^Bak^-/-^* mouse embryonic fibroblasts (MEF) with or without F-BAK^G51C^, as in Figure S2a, and membrane fractions incubated with cBID, the 7D10 antibody IgG (~150 kD), or the smaller 7D10 scFv (~25 kD). For immunoprecipitation, a mouse antibody (14-36) that recognizes activated BAK was added, and samples immunoblotted for BAK (aa23-38, upper panels) or for the FLAG epitope present in F-BAK^G51C^ only (lower panels). Schematic (right) illustrates that the smaller size of the 7D10 scFv. Note that the beads used in the immunoprecipitation would bind the 14-36 antibody but not the 7D10 scFv. Data are representative of two independent experiments.

**Supplementary Figure 6. Animation of the hit-and-run interaction involved in autoactivation by BAK**

An activated BAK monomer (right) binds to non-activated BAK (left) via a transient BH3:groove interaction to trigger a series of conformation changes: α1-dissociation, α2 (BH3) exposure, and separation of core (α2-α5) from latch (α6-α8). Following disconnection of the two activated monomers, they can then associate via a reciprocal BH3:groove interaction to form stable homodimers. Note that α9 is inserted as a transmembrane domain into the mitochondrial outer membrane, and that some of the conformation changes may occur in concert but are shown sequentially for clarity. Helices are colour-coded (1, blue; 2, red; 3, cyan; 4 light pink; 5 green; 6, purple; 7, magenta; 8, orange; 9, yellow). Animation of other BCL-2 family protein interactions is available at https://www.wehi.edu.au/wehi-tv/apoptosis-venetoclax.

**Supplementary Table 1. Guide to paired variants of BAK and BAX used to test for autoactivation**

*^α^BAK is human with endogenous cysteines residues at C14 and C166 (except where indicated)*

*^β^BAX is human with endogenous cysteines residues at C62 and C126 (except where indicated)*

*^χ^F denotes FLAG-tagged protein*

*^δ^For details, see Methods. Also reviewed in^45^*
