# Supplementary figures and images for: Robust autoactivation for apoptosis by BAK but not BAX highlights BAK as an important therapeutic target
Source: Cell Death Dis. 2020 Apr 23;11(4):268. doi: 10.1038/s41419-020-2463-7 (PMC7181796; doi:10.1038/s41419-020-2463-7)

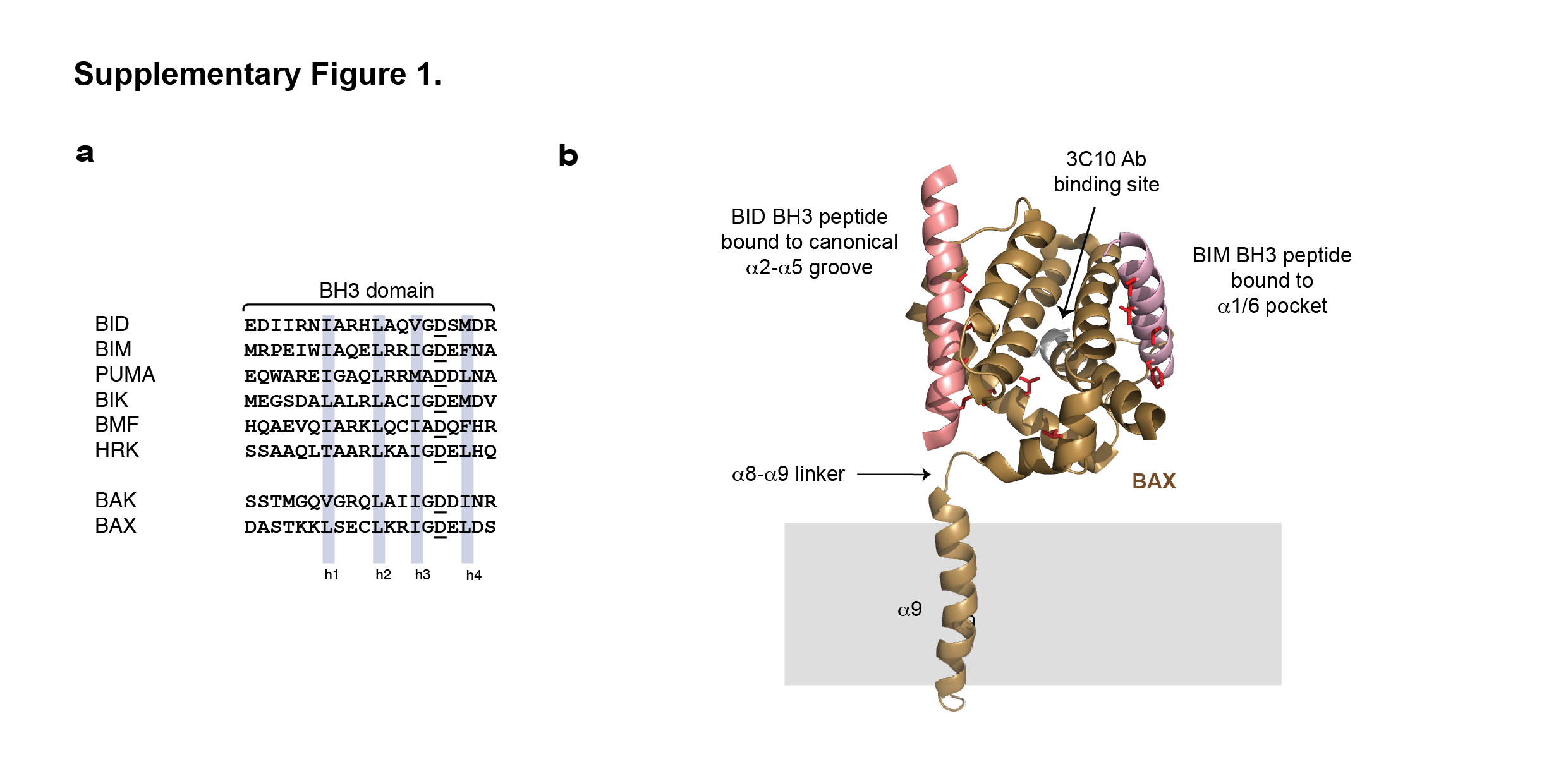

Supplement: Supplementary file 2 — Supplementary Figure 1 [file 41419_2020_2463_MOESM2_ESM.png]

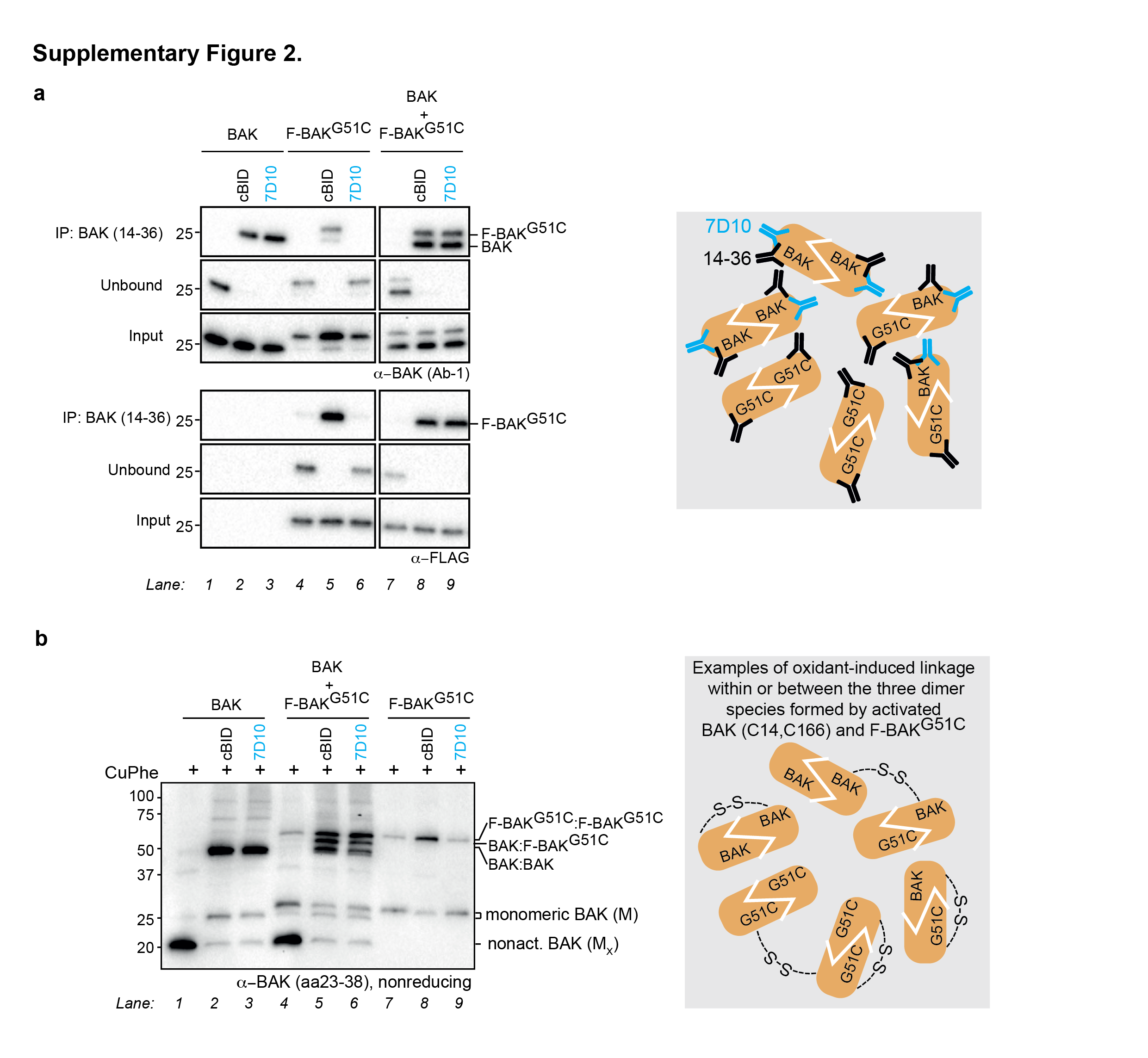

Supplement: Supplementary file 3 — Supplementary Figure 2 [file 41419_2020_2463_MOESM3_ESM.png]

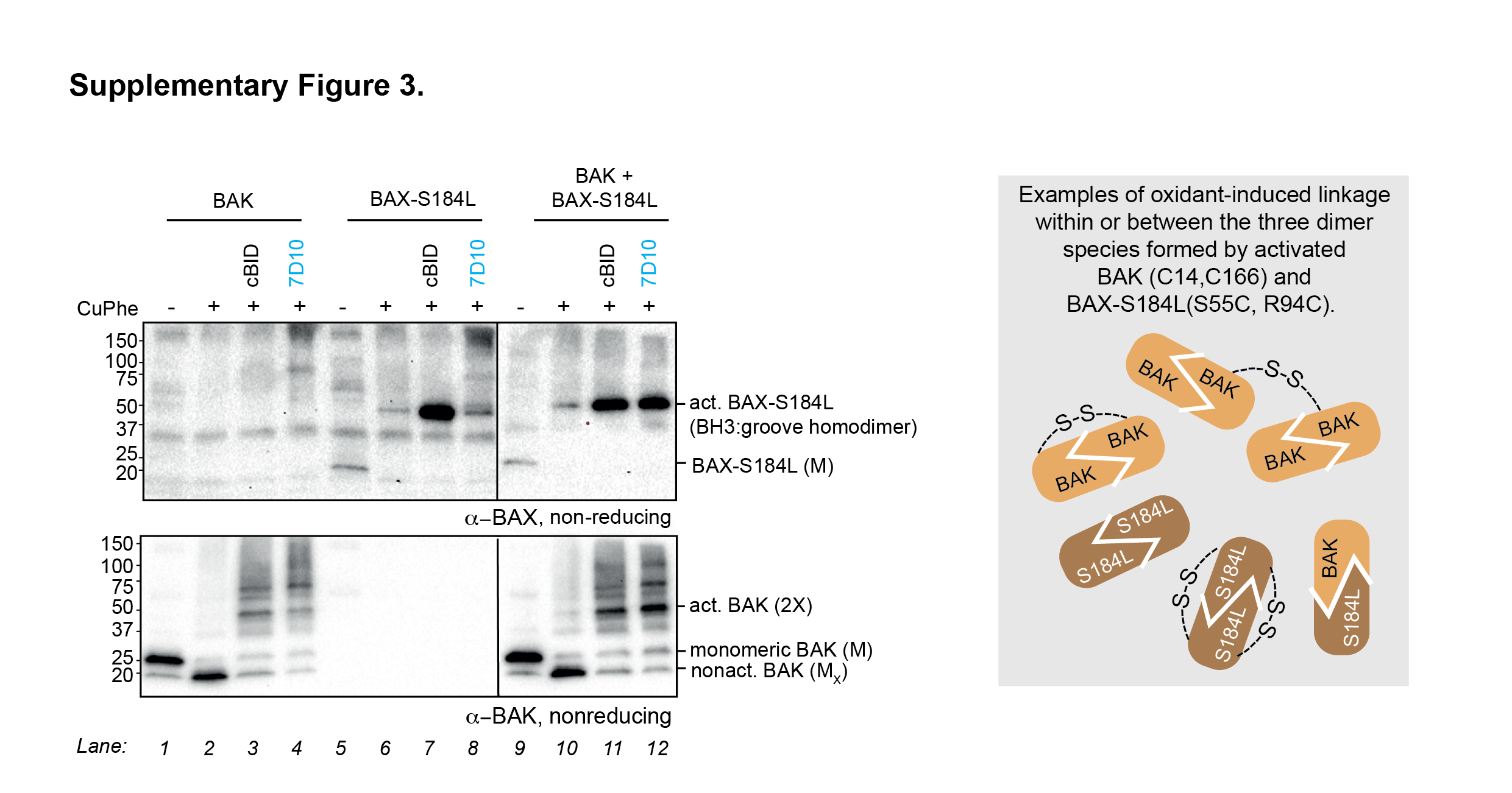

Supplement: Supplementary file 4 — Supplementary Figure 3 [file 41419_2020_2463_MOESM4_ESM.png]

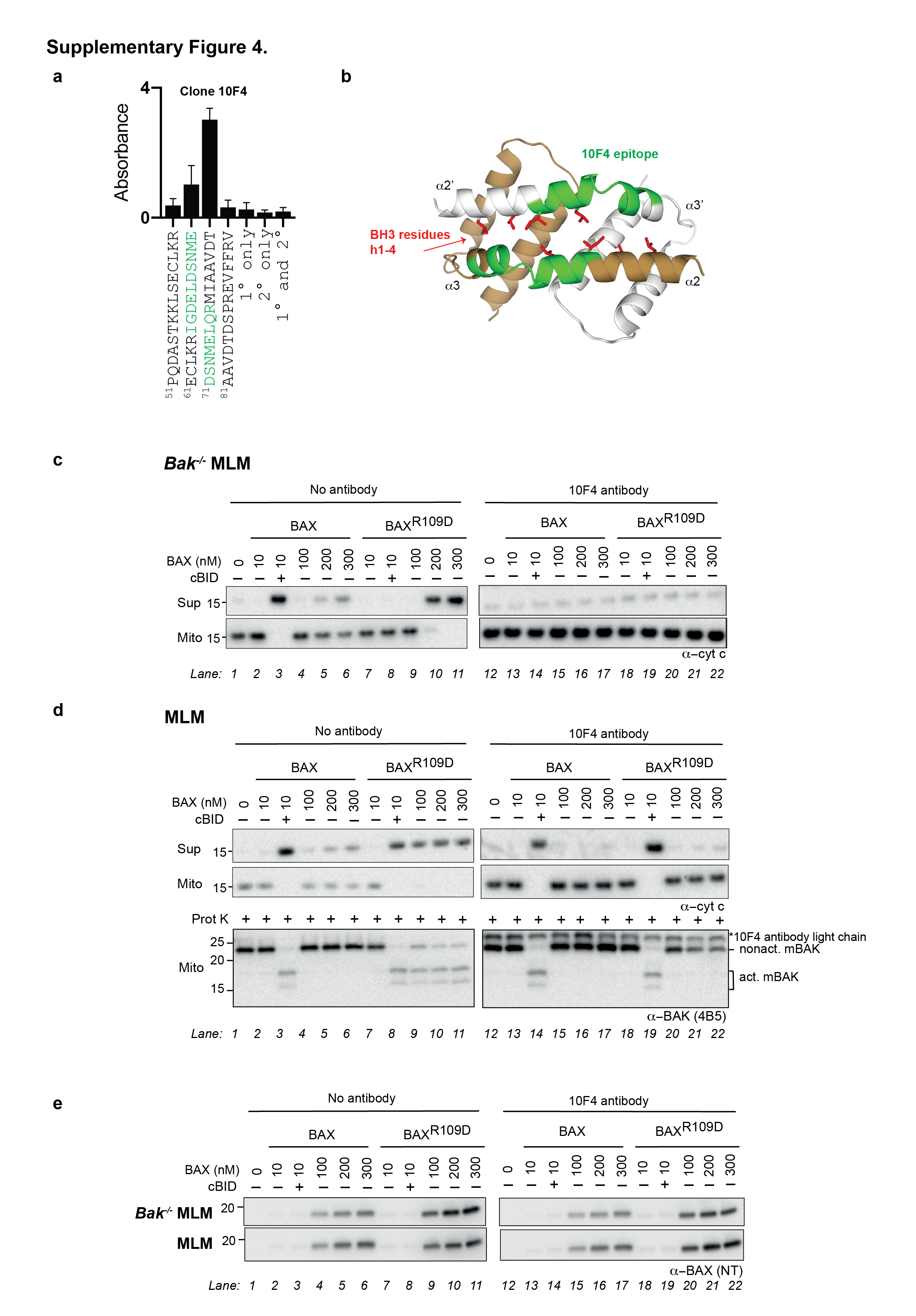

Supplement: Supplementary file 5 — Supplementary Figure 4 [file 41419_2020_2463_MOESM5_ESM.png]

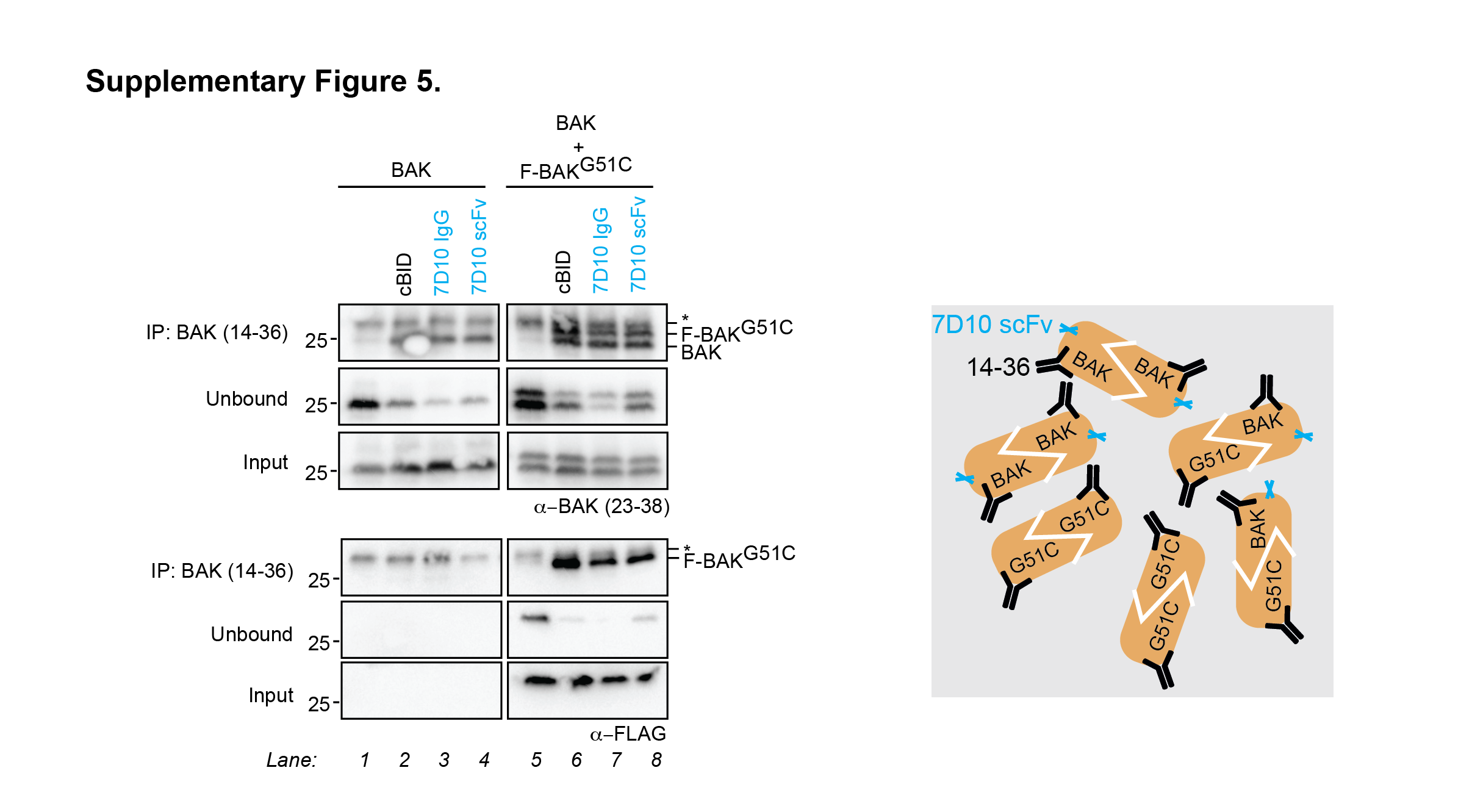

Supplement: Supplementary file 6 — Supplementary Figure 5 [file 41419_2020_2463_MOESM6_ESM.png]
